# Supplementary material for: The association between a rotator cuff tendon tear and a tear of the long head of the biceps tendon: Chart review study
Source: PLoS One. 2024 Mar 11;19(3):e0300265. doi: 10.1371/journal.pone.0300265 (PMC10927094; doi:10.1371/journal.pone.0300265)
Supplement: Attachment — Submitted filename: Response to Reviewers.docx [file pone.0300265.s001.docx]

| - Thanks for reviewing the manuscript and for your helpful comments. The editor’s and reviewers’ comments were reviewed and addressed below in the Response to Reviewers letter as requested, as well as all changes made in the revised manuscript as requested. | | |
| --- | --- | --- |
| **Comments** | **Section** | **Response** |
| **Journal Requirements:** | |  |
| 1. Please ensure that your manuscript meets PLOS ONE's style requirements, including those for file naming. The PLOS ONE style templates can be found at  <https://journals.plos.org/plosone/s/file?id=wjVg/PLOSOne_formatting_sample_main_body.pdf> and  <https://journals.plos.org/plosone/s/file?id=ba62/PLOSOne_formatting_sample_title_authors_affiliations.pdf> | **Manuscript** | - The manuscript was amended according to the PLOS ONE style templates. |
| 2. Note from Emily Chenette, Editor in Chief of PLOS ONE, and Iain Hrynaszkiewicz, Director of Open Research Solutions at PLOS: Did you know that depositing data in a repository is associated with up to a 25% citation advantage (<https://doi.org/10.1371/journal.pone.0230416>)? If you’ve not already done so, consider depositing your raw data in a repository to ensure your work is read, appreciated and cited by the largest possible audience. You’ll also earn an Accessible Data icon on your published paper if you deposit your data in any participating repository (<https://plos.org/open-science/open-data/#accessible-data>). |  | - The data set was uploaded in HARVARD Dataverse with the following access link: <https://doi.org/10.7910/DVN/F3VD46> |
| 3. Your ethics statement should only appear in the Methods section of your manuscript. If your ethics statement is written in any section besides the Methods, please delete it from any other section. |  | - The ethical statement is written only in the Methods section (Line# 129-132), and it was deleted from any other section in the manuscript. |
| 4. We note that you have indicated that there are restrictions to data sharing for this study. PLOS only allows data to be available upon request if there are legal or ethical restrictions on sharing data publicly. For more information on unacceptable data access restrictions, please see <http://journals.plos.org/plosone/s/data-availability#loc-unacceptable-data-access-restrictions>. |  | - The data set was uploaded in HARVARD Dataverse with the following access link: <https://doi.org/10.7910/DVN/F3VD46> |
| Before we proceed with your manuscript, please address the following prompts:  a) If there are ethical or legal restrictions on sharing a de-identified data set, please explain them in detail (e.g., data contain potentially identifying or sensitive patient information, data are owned by a third-party organization, etc.) and who has imposed them (e.g., a Research Ethics Committee or Institutional Review Board, etc.). Please also provide contact information for a data access committee, ethics committee, or other institutional body to which data requests may be sent.  b) If there are no restrictions, please upload the minimal anonymized data set necessary to replicate your study findings to a stable, public repository and provide us with the relevant URLs, DOIs, or accession numbers. For a list of recommended repositories, please see <https://journals.plos.org/plosone/s/recommended-repositories>. You also have the option of uploading the data as Supporting Information files, but we would recommend depositing data directly to a data repository if possible.  We will update your Data Availability statement on your behalf to reflect the information you provide. |  | - The data set was uploaded in HARVARD Dataverse with the following access link: <https://doi.org/10.7910/DVN/F3VD46> - The Data Availability Statement was updated as following: “All data generated or analyzed during the current study are included in this article. The data is available at <https://doi.org/10.7910/DVN/F3VD46>. Publishing the data requires prior approval from the Institutional Review Board (IRB), King Abdullah International Medical Research Center (KAIMRC), Ministry of National Guard, Health Affairs, P.O. Box 3660, Riyadh 11481, Saudi Arabia. Mail Code: 1515. Telephone Number: +966-11-429-4444, Extension Number: 94456. Email: [IRB@mngha.med.sa](mailto:IRB@mngha.med.sa) ” (Line#557). |
| **Reviewers' 1 Comments:** | |  |
| The manuscript entitled “The Association Between a Rotator Cuff Tendon Tear and a Tear of the Long Head of the Biceps Tendon: Chart Review Study” by Alraddadi et al. addresses an extremely interesting and original topic concerning the association between an injury of the long head of the biceps tendon and the tear of the rotator cuff tendon evaluated on a large group of patients. In general, the provided text appears clear and concise, presenting key findings from the study. The language is straightforward and effectively communicates the main objective and results. The abstract clearly presents the study, the introduction is well-written and allows to have a comprehensive vision of the topic. The conclusions are clear and concise. | **The Manuscript** | **We appreciate your review and your insightful feedback on the manuscript.** |
| In my opinion, the work could be further enriched in the materials and methods section. This part could be implemented by dividing the different sections into titled subparagraphs for better reader understanding. The same approach could also be used for the results section. | **Materials and Methods**  **Results** | - Titled subparagraphs were added to the Materials and Methods and Results sections as recommended. |
| Please check that table 4 is complete and standardize table 5 by adding the statistic in the table. | **Results** | - The data in Table 4 has been verified and confirmed. However, the number of specimens in the cells of the first and second rows did not meet Cochran's criteria for accepting the results of the Chi-Square test. Consequently, a note was included underneath the table to enhance clarity for the reader. - The statistics of Table 5 were added as recommended by the 1^st^ Reviewer. |
| **Reviewer’s 2 comments** | |  |
| - The uniqueness of the current study is called into doubt because the purpose and goals of this investigation have already been made clear in a number of well-published studies as the authors mentioned in the “Discussion” (Lines 224-225) (references number 9,34-37 in the current manuscript). Like the current study, previous studies proved a direct positive association between intensity of the RC tears and LHBT involvement (references number 35 and 37 in the current manuscript). Also, one of the findings in the current study is a higher incidence of LHBT tears in shoulders with multiple RC tendon tears, rather than a single tendon tear.   Again, this is not anew finding and previously proved in previous studies. |  | **We appreciate your review and your insightful feedback on the manuscript.**   - The study stated in the discussion section that: “This study confirmed that there is an association between RC and LHBT tears” (Line#358). In addition, the current study highlighted the uniqueness of the current study in the limitation section: “A further advantage of this study was the removal of individuals with significant injuries, as the objective was to monitor the neutral degenerative process in the absence of physical harm” (Line# 522). Thus, the current study found that no shoulder without RC tear had LHBT tear. In contrast, the previous studies investigated the relationship between the RC and LHBT tears included shoulders with traumatic injuries. - To improve this part, the following sentence was also revised in the limitation paragraph: “The most important finding of this study was that it confirmed the relationship between RC and LHBT tears, as reported in previous studies.” (Line#528). |
| **Abstract**   - Line 13: “..due to an rotator cuff ..” “an” should be deleted - Line 20: The meaning of the abbreviation “RC” was not previously mentioned. As a rule, with the abstract considered a spate entity, the abbreviation of any word should be mentioned on its first use, then the abbreviation only should be used afterword. | **Abstract** | - “an” was deleted as recommended by the 2^nd^ reviewer and checked throughout the manuscript. - The abbreviations “RC” and “LHBT” were identified and used throughout the abstract. |
| - The introduction section is lengthy. It needs to be shortened. Kindly focus on three elements of introduction.   a. What is known about the topic? (Background)  b. What is not known? (The research problem)  c. Why the study was done? (Justification) | **Introduction** | - The introduction was shortened, when feasible, to enhance its focus on the study's aim. The deleted sections were marked with a crossed line in the revised manuscript, and the references were updated correspondingly. |
| - Lines 113-114: What is the meaning of this sentence: “The presence of one tendon partial tear was the commonest pattern of RC partial and full thickness tears as it was detected in 77% and 77.7% of patients, respectively.”? | **Results** | - The sentence was rephrased as follow: “One tendon tear was the most common pattern of RC partial and full-thickness tears, seen in 77% and 77.7% of patients, respectively.” (Line#185). |
| - Line 125: “Acromioclavicular osteoarthritic changes” should be corrected to “Acromioclavicular joint osteoarthritic changes | **Results** | - The mentioned statement was corrected as recommended (Line#199). |
| - Line 139: “tor” is a topographical error should be corrected to “torn” | **Results** | - The topographical error was corrected (Line# 217). |
| - Line 141: “An examination of the association between the LHBT and RC tears is presented in Table 3.” Should be corrected to “The association between the LHBT and RC tears is presented in Table 3.” | **Results** | - The mentioned sentence was revised as recommended (Line#223). |
| - First paragraph of the “Discussion” should summarize the results of the study | **Discussion** | - The first paragraph of the study was revised to summarize the study’s results (Line#303). |
| - “Discussion” is too long and should be shortened. | **Discussion** | - The discussion was shortened when feasible without compromising its strength or quality. The deleted sections were indicated with a strikethrough in the revised manuscript, and the references were appropriately amended. |
